# A Wearable System for Gait Training in Subjects with Parkinson's Disease

**DOI:** 10.3390/s140406229

**Published:** 2014-03-28

**Authors:** Filippo Casamassima, Alberto Ferrari, Bojan Milosevic, Pieter Ginis, Elisabetta Farella, Laura Rocchi

**Affiliations:** 1 Biomedical Engineering Unit, DEI, University of Bologna, 40136 Bologna, Italy; E-Mails: alberto.ferrari@unibo.it (A.F.); l.rocchi@unibo.it (L.R.); 2 Micrel Lab, DEI, University of Bologna, 40136 Bologna, Italy; E-Mails: filippo.casamassima@unibo.it (EC); bojan.milosevic@unibo.it (B.M.); 3 Neuromotor Research Group, Department of Rehabilitation Sciences, KU Leuven, 3001 Leuven, Belgium; E-Mail: pieter.ginis@faber.kuleuven.be; 4 E^3^DA Unit, ICT Center, Fondazione Bruno Kessler, 38123 Trento, Italy; E-Mail: efarella@fbk.eu

**Keywords:** wearable, body area networks, motor disorders, rehabilitation, Parkinson's disease, audio-feedback, gait analysis

## Abstract

In this paper, a system for gait training and rehabilitation for Parkinson's disease (PD) patients in a daily life setting is presented. It is based on a wearable architecture aimed at the provision of real-time auditory feedback. Recent studies have, in fact, shown that PD patients can receive benefit from a motor therapy based on auditory cueing and feedback, as happens in traditional rehabilitation contexts with verbal instructions given by clinical operators. To this extent, a system based on a wireless body sensor network and a smartphone has been developed. The system enables real-time extraction of gait spatio-temporal features and their comparison with a patient's reference walking parameters captured in the lab under clinical operator supervision. Feedback is returned to the user in form of vocal messages, encouraging the user to keep her/his walking behavior or to correct it. This paper describes the overall concept, the proposed usage scenario and the parameters estimated for the gait analysis. It also presents, in detail, the hardware-software architecture of the system and the evaluation of system reliability by testing it on a few subjects.

## 1. Introduction

Parkinson's disease (PD) is a neurodegenerative disorder characterized by specific motor impairments, which include tremor, bradykinesia (*i.e.*, slowness of movement), rigidity (*i.e.*, resistance to externally imposed movements) and postural instability PD is caused by the deficiency of a neurotransmitter, called dopamine, in the brain, specifically in the basal ganglia, a group of nuclei involved in movement control. The complex neurological degeneration in PD particularly causes a severe difficulty in motor planning, action and execution of non-attention demanding tasks (automaticity) [1,2]. Since PD is a neurological disorder with progressive motor disability over time, the role of rehabilitation has been questioned for many years [3]. However, in the past two decades, there has been evidence from research studies demonstrating the ability of motor learning in PD, as well as improvements in motor function as a result of training [4]. In fact, it has been shown that pathways involving the basal ganglia in PD may be capable of plasticity, and their activity patterns may be partly corrected with appropriate intensive training [5–7]. These results are opening up new paths to extend the concept of motor learning and rehabilitation to neurological diseases and to PD in particular [7,8]. This evidence is at the basis of the EU-funded research project, CuPiD (closed-loop system for personalized and at-home rehabilitation of people with Parkinson's disease, http://www.cupid-project.eu/), in the context of which, the present study has been conceived and developed.

Motor learning is classically defined as a set of processes associated with practice or experience [4], leading to relatively permanent changes in the capability for movement. Although the rates of motor learning may be slower in PD, recent evidence highlights that PD patients can acquire motor skills and improve their motor performance with sustained personalized training [5,6,9].

Gait disturbances are very common factors in patients with Parkinson's disease, causing severe disabling consequences and representing one of the major determinants for the quality of life of these patients. PD gait is characterized by a difficulty with the internal regulation of stride length and step clearance and increased gait variability [1,10]. Associated disturbances include a forward-flexed trunk, inadequate flexion at the ankle and knee, insufficient heel strike, postural instability and asymmetric stride. In addition, patients show problems in maintaining rhythmicity and the amplitude of the gait [11,12].

The use of additional sensory information was found to be an appropriate tool to optimize motor learning in PD [4,13]. Real-time feedback on motor performance using signals from the patient's own body is a technique that helps to improve neuro-motor performance [14,15]. It works by adding artificial sensory information (sensory augmentation or substitution) and involving cognitive components. The working principle is to take information from specific body signals, code it into appropriate sensory signals and feed these back to the user in real time [9,16,17].

Motor learning through augmented sensory information has been used with important results in the rehabilitation and exercising of gait, step and dynamic repetitive movements. Adopting external cues, such as rhythmic auditory stimulation (RAS), appears to enhance motor performance, in particular in the context of stepping and gait. Studies involving RAS are based on open-loop systems, *i.e.*, the cues are administered without taking into account the actual performance of the patient in real time [4,18].

In the present study, we aim to advance from the state-of-the-art using open-loop approaches (feedforward responding to a movement reference) by adopting a closed-loop approach (feedback, responding to information about movement performance) to enhance motor learning in specific tasks for patients with PD. In particular, the contribution of the current work is to present a system for gait evaluation and rehabilitation, to be used in a daily life setting. The system is based on real-time computation of gait features, which are fed back to the user with the aim of helping him/her execute the most effective gait pattern, stimulating the motor learning process. To achieve this goal, the system employs a fully portable and wearable architecture, *i.e.*, a Wireless Body Sensor Network (WBAN), based on Inertial Measurement Units (IMUs), connected with a smartphone used as a portable processing platform. A preliminary validation of the proposed approach is presented using data from multiple PD patients.

## 2. System Concept and Design

The system concept and design principles were built into a patient-specific framework, with the aim of dealing with specific scientific and technological challenges, including:

- Automatic and real-time computation of gait features from an easy-to-use system set-up;
- The definition of rules for real-time feedback restitution;
- Identification and optimization of the type of feedback (reward/negative; continuous/discrete).

The system's use was planned to be very easy, possibly exploitable in a domestic setting and based on a very simple sensors set-up. The information returned to the user during training aims at reflecting the nature of instructions normally provided to the patient by the therapist during traditional rehabilitation trials, potentially with higher precision and reliability than human instructions. Following clinicians' specific experience, a literature review and feasibility criteria [19], the gait features listed in Table 1 were defined as key characteristics, especially for PD, and as good candidates as variables to be trained.

In summary, the system and its training possibilities are designed to allow the functional goals of improving the rhythm and amplitude of the gait, increasing vigorousness, decreasing the asymmetry of movements and preventing shuffling and forward inclination. Spatio-temporal gait parameters, such as cadence, gait speed, *etc.*, provide the information needed to monitor the performance of a subject's walk and an accurate measure of the overall efficacy of the locomotor function. In addition, beside the computation of typical gait features related to the lower limbs, the system was intended to monitor any abnormal forward inclination of the trunk. This is a strong disabling symptom in some PD patients with important effects on gait efficacy [20,21].

In the scenario of use, the subject is trained to adopt and maintain a correct trunk posture and correct gait patterns, during short periods of the day, via real-time detection and subsequent feedback restitution of his/her trunk kinematics and gait performance. In this way, patients can, at least partially, re-learn the correct gait pattern and trunk posture or develop strategies to overcome their disability [4,8].

**Figure 1.**
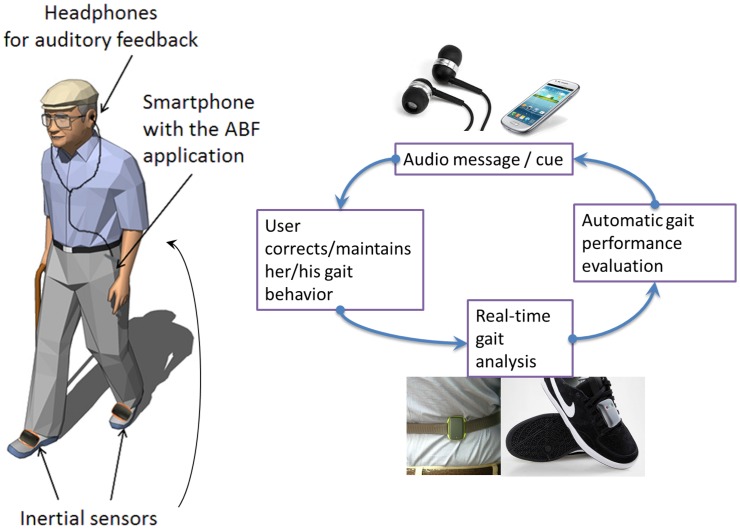
Scheme of the scenario for the use and the components of the system.

To be able to compute the aforementioned features, minimizing the number of sensors, but maximizing the reliability, wearability and flexibility of the system, a set-up based on three sensors and one processing unit (a smartphone) has been selected. Two sensors are mounted on the shoes and a third one is mounted on the trunk (Figure 1). The feedback is provided using vocal messages delivered via headphones.

The therapeutic plan may follow a progressive adaptation and modification of the combinations of variables monitored by the system. Specifically, as soon as a specific gait feature has been trained to a certain extent, the biofeedback system will be tuned to focus on a different feature.

The training scenario at the basis of this design includes unconstrained gait (with possible stops, turning, road-crossing, *etc.*) to be performed in combination with the biofeedback system. The long-term goal is to train people to maintain their own optimal gait for the entire duration of the trial (possibly lasting around 30 min). The system presented, and specifically developed, in this study is essentially composed of three elements:

1. The inertial sensor nodes;
2. An Android mobile phone;
3. The intelligence on-board able to compute gait features and to propose real-time feedback.

Hardware (and related firmware) components will be presented in the next section, while the application and the related algorithms will be introduced in the following one.

## 3. System Architecture

**Figure 2.**
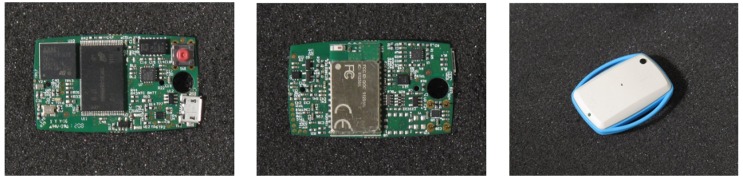
The EXLs1 sensing unit.

In the present study, a smart sensor platform based on IMUs and specifically designed in the context of the EU-funded project, CuPiD, was customized and used for the first time. The sensing unit, EXLs1 (by EXEL, Bologna, Italy) (Figure 2) is an open and versatile alternative to available commercial solutions.

Each node includes an STM32F103 microcontroller, based on the ARM Cortex™-M3 core running up to 72 MHz. A combination of Micro-Electromechanical Systems (MEMS) sensors produced by STMicroelectronics (accelerometer, gyroscope and magnetometer) is embedded in the sensor nodes, while communication with external devices is performed with a standard Bluetooth module (STM SPBT2532C2.AT). The hardware configuration is completed by a 1 Gb NAND FLASH memory for local data storage and a Li-ion battery. The sensor nodes have a standard micro-USB port, which is used to recharge the battery. This can be easily performed with a standard smartphone charger, compatible with all Android phones, or with a docking station provided by the manufacturer.

The firmware, specifically implemented for the present study, was designed to optimize the hardware components and for allowing the sensing units to work in different modes: data streaming via Bluetooth; data logging in the internal FLASH memory; with an on-board processing if necessary (signal pre-processing or orientation estimation, as described in the following section).

### 3.1. Power Management

To improve the usability of the sensing units, in particular in an unsupervised environment (the patient's house or in daily life), careful energy management of hardware and firmware resources is necessary to increase their battery lifetime. One of the approaches that can be exploited to reduce the power consumption of a sensor node is the duty cycling (DC) policy, which can be applied even when accuracy requirements are strict, and it is based on a sleep/wake-up scheduling protocol [22]. This kind of approach has been pursued for our nodes. To apply a DC policy, dynamic power management techniques are used to decrease the energy consumption by selectively placing idle components into low-power states. The scheduling protocol is defined taking into account the application's sampling rate requirements and the cost of the transitions between the different power states [23].

Since the radio transceiver significantly affects the power consumption of the node when data are transmitted, the Bluetooth's Sniff mode was employed to reduce the power consumption. It is used only when advantageous, since we found that for high transmission rates, the Sniff mode uses more energy than the normal operating mode [24].

**Figure 3.**
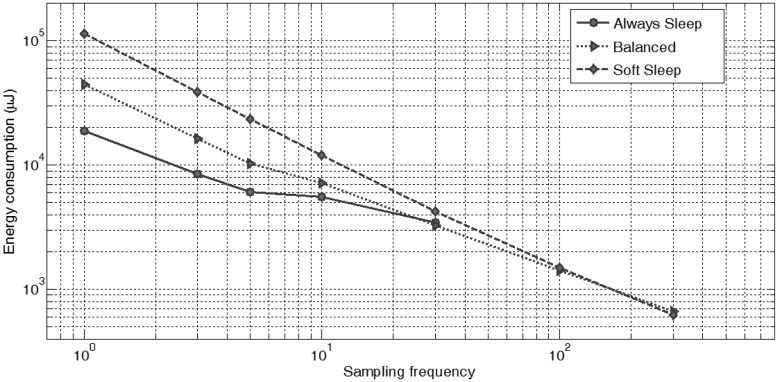
Energy consumption evaluations of the node's overall energy consumption (per sample).

The use of an appropriate policy greatly reduces the energy cost: our results show that the reduction can be 7.5% when sampling at 300 Hz, 30% when sampling at 30 Hz and even up to 300% if sampling at 3 Hz, as shown in Figure 3 and demonstrated in detail in [24].

In the typical application scenario used for gait analysis, sensors were sampled at 100 Hz, and calibrated sensor data was streamed via Bluetooth, achieving an average energy consumption of 29.8 mA and a battery lifetime of 6 h, 12% up compared to the baseline case.

### 3.2. Networked Operation

In applications, such as the ones described in the present study, the synchronization between different sensing units of the Bluetooth network is essential. To this aim, it is crucial that data collected from the various nodes are synchronized and share a common time base.

The time synchronization problem for wireless networks has been extensively studied in the literature over the last two decades, and yet, there is no specific synchronization scheme capable of guaranteeing a high order of accuracy with great scalability, independent of the topology and application [25]. Bluetooth synchronization protocols based on the exchange of explicit synchronization messages suffer from highly variable messages latencies [26]; as a consequence, in our case, the shared network clock was used instead [27]. All Bluetooth devices are equipped with a 28-bit counter clock with 0.3125-ms resolution and a mandatory maximal drift of ±20 ppm. During the establishment of the network, the difference between the local Bluetooth clock and the clock of the different devices is acquired. This information is then used to form a common time base for all the devices and to synchronize the obtained sensor data.

### 3.3. Sensing Nodes Calibration and On-Board Orientation Estimate

For the correct use of sensor data, a rapid and reliable calibration procedure was defined. Even if the different sensors were factory calibrated, an additional calibration step was required to compensate for possible misalignment during their assemblage into the final device. Calibration methods for inertial and magnetic sensors are well documented [28,29]. For the calibration procedure, six static poses and rotations of the device around its three main axes are considered, and the data is processed to obtain a calibration matrix and an offset vector for each sensor.

To evaluate the performance of the sensor node, we compared it with a commercial high-end solution, such as the MTw sensor platform (by Xsens, Enschede, Nedherlands). For this purpose, we collected raw data from the two platforms in a static condition for 10 s, which was used to compute the sensors' noise in terms of the standard deviation around the mean values. The results are in Table 2, and they show a very similar performance for the two platforms, reporting the standard deviations of each axis for the three sensors.

IMU sensor data can be used to estimate the orientation of the device, providing the implicit orientation of the body segment on which they are mounted. Being one of the most common types of information to be extracted from an inertial body sensor network, the orientation estimation is calculated directly on-board the devices.

To avoid the creation of a communication and computation bottleneck on the host device, each employed node can compute its own orientation by exploiting the embedded microcontroller, and achieve better energy efficiency avoiding to send all the sampled data to the host device.

State-of-the-art algorithms for the estimation of orientation from IMU sensor data were optimized for the EXLs1 sensor node, including the Kalman filter [30], its extended variation [31] and recently developed complementary filters [32,33]. These different algorithms were implemented and are available on the EXLs1 sensor platform, where they were directly compared. All the algorithms were optimized to run in real time on the resource-constrained embedded MCU, and their performance was compared in terms of accuracy, computational cost and energy efficiency. The Kalman Filter approach resulted in being the most flexible one and was therefore the preferred choice for our application.

The precision achieved on the MCU was tested against the MATLAB implementation, which uses 64-bit double precision values and was computed on a PC. For this purpose, both the sensor data and the computed quaternions were sent from the device to the PC, to allow for the elaboration of the same stream of data on the two platforms. The difference between the orientation computed on the two platforms is negligible, having an RMSE lower than 0.004 degrees for roll, pitch and yaw angles.

**Figure 4.**
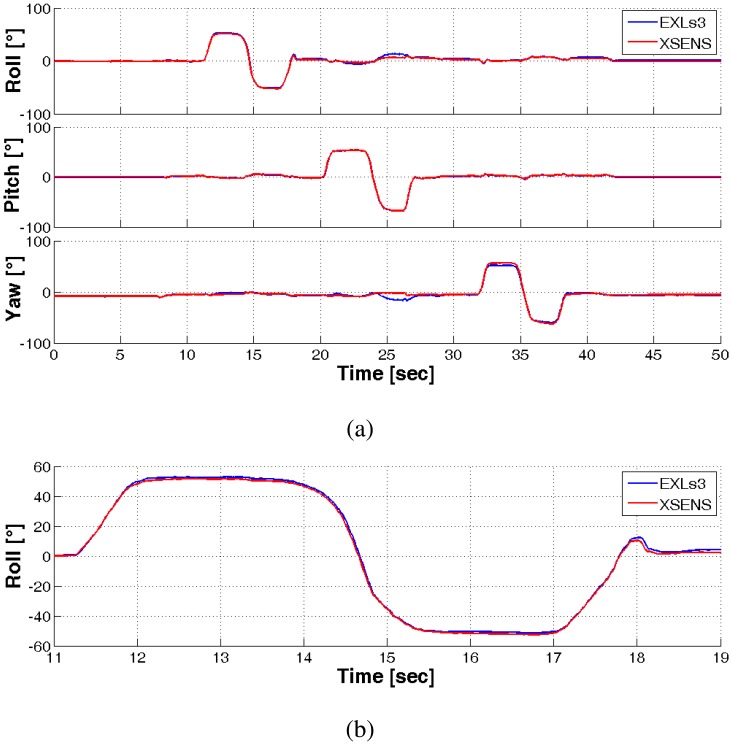
(**a**) Comparison of orientation estimation as computed by Xsens MTw (red) and EXLs1 (blue) sensor nodes using the Kalman filter. (**b**) Zoom of the roll plot.

A further sensor validation check was performed in terms of orientation estimation, comparing the two sensing platforms. In this case, two nodes were attached to each other and rotated in air, while logging the computed orientation from the systems. The result of 50 s of computed data is shown in Figure 4. The difference between the two estimations is minimal, even if the two nodes were manually aligned, with an RMSE of 1.818, 0.594 and 2.941 degrees for the three estimated angles.

## 4. Algorithms and Real-Time Feedback for Gait Rehabilitation

Overall, the functional goal of the system entails the improvement of the gait's rhythm and amplitude, the reduction of asymmetry and the correction of unstable posture assumption by means of recurrent audio feedback provided in real time during unconstrained walking. Feedback restitution is performed by a logic flow of states and conditions able to tutor the patient in maintaining the gait pattern and performance at a specific target set by a clinician. Furthermore, the frequency and total amount of messages provided to the patient are adaptively controlled, to avoid saturation effects. Besides, the degree of difficulty of the task is automatically tuned to be challenging, but not too demanding. Finally, vocal messages, encoded by a text-to-speech application, have been formulated from clinicians to stimulate those motor adjustments usually prompted during rehabilitation sessions in clinical environments.

In order to implement these functions, an Android mobile phone (equipped with a Bluetooth connection capable to run Android 2.3) has been adopted to manage the network, send commands and receive data from the sensor nodes. The intelligence on-board the system encompasses two main functions:

- soft real-time automatic identification of gait parameters;
- soft real-time feedback restitution to the user.

The estimation of gait spatio-temporal parameters concerns, first of all, the segmentation of walking into gait cycles, which entails the detection of the events, initial contact (IC) and foot-off (FO). A new online automatic algorithm was developed to detect in real time the IC and FO by processing the angular velocity along the medio-lateral axis of the IMUs, which are rigidly attached to the patient's shoes. Once these events are detected, it is possible to determine the gait spatio-temporal parameters, such as cadence, gait asymmetry or step length by means of *ad hoc* signal filtering techniques (see Sections 4.1 and 4.2).

Based on clinical practice and feasibility criteria [34], the following gait parameters were identified as the most clinically meaningful, namely: cadence, step length, trunk posture, gait speed, gait asymmetry and clearance. The real-time computation of these parameters was implemented into the application.

### 4.1. Soft Real-Time Gait Events Detection

For the automatic detection of gait events using inertial sensors, several approaches have been proposed in [35,36]. They differ in terms of sensor locations and the processing of accelerations and/or angular velocities to estimate IC and FO events [35].

In this context, it was decided to keep the number of IMUs as limited as possible in order to: (1) ergonomically improve the ease of use, decreasing the required time to attach sensors on body segments and reducing the hindrance to natural walking; and (2) technically reduce the power and memory requirements of the central processing unit.

**Figure 5.**
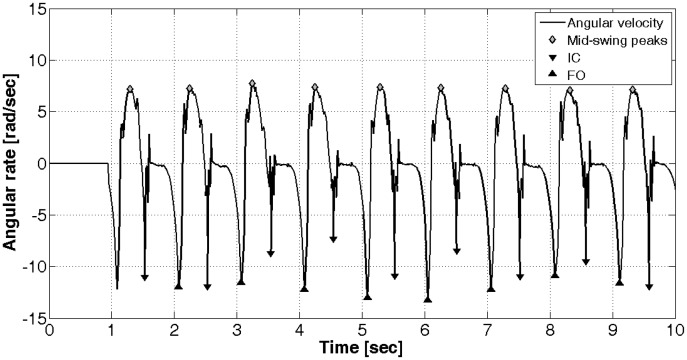
Angular velocity and the detected initial contact (IC) and foot-off (FO) events.

Among all the different methods proposed in the literature for detecting IC and FO, the one from Lee *et al.* [37] has been used as the starting point to implement a novel method conceived specifically to deal with our application scenario. This new algorithm is able to determine IC and FO by processing the angular velocity signal arising along the medio-lateral axis of the foot. In particular, during each single gait cycle, the foot rotates alternatively clockwise and anticlockwise around the ankle joint, hence allowing the possibility of segmenting the gait cycle in its different temporal phases. The implemented algorithm first identifies all positive peaks (feet in anticlockwise rotations by looking at a patient walking from his/her right side) associated with mid-swing events [36], then, within each couple of mid-swing peaks, finds out the first negative peak (feet clockwise rotations), being the IC, and the second (in time) negative peak, being the FO (see Figure 5).

The identification of the positive and negative peaks is based on the MATLAB built-in function, *findpeaks()*. Gait speed and pattern and the presence of flat or uneven terrain produce peaks differing in terms of amplitude and sharpness. In order not to lose sensitivity and specificity in IC and FO identification, the internal parameters of the findpeaks function (minimum peak height, minimum peak separation in time, minimum height difference and number of peaks) are automatically tuned from the algorithm on the basis of a dataset of healthy subjects and patients walking at different speeds (ranging from 0.4 m/s to 1.8 m/s), with different patterns outdoor and indoor (see Section 4.4 for the accuracy of the method).

From the knowledge of the IC and FO instants, it is then possible to easily define cadence, duration of stride, stance, swing phases and all other gait temporal parameters.

### 4.2. Soft Real-Time Step Length Estimation

Pedestrian dead-(for deduced) reckoning allows one to estimate the feet positions in real time from the use of two sensors attached to the feet [38]. Dead-reckoning is the process of calculating one's current position by using a previously determined position, or fix, and advancing that position based upon known or estimated speeds over elapsed time and the course. Theoretically, considering the accelerometer as a linear motion sensor and the gyroscope as a rotation sensor, by double integration of the acceleration of an IMU (e.g., attached to the shoe), it is possible to track the position in time of a subject's walking in any environment. However, in practice, this is an unfeasible operation, since the orientation-dependent gravitational component is not straightforwardly separable from the inertial acceleration, and even minor drift internal to the gyroscope or the accelerometer (being single-/double-integrated) causes errors that increase cubically [39].

Pedestrian dead-reckoning allows continuous computing of the position, orientation and velocity of a subject's motion by implementing additional filtering based on human walking constrains. When a person walks, his/her feet are periodically in a stance-stationary phase in which the entire foot is in contact with the floor and the body shifts forward by rigidly pivoting the lower limb on the ankle joint (the second rocker phase of the gait cycle). During this stance stationary phase, it is possible to draw an advantage from the condition of having the foot at zero velocity, hence filtering errors accumulating in the integration of acceleration and correcting the position estimated up to the second rocker phase. In other words, zero-velocity updates (ZUPTs) act as pseudo-measurements, allowing for the reduction and limitation of position errors. The pedestrian dead-reckoning algorithm implemented in this study was developed starting from the method proposed by Nilsson *et al.* [38].

A range of detectors aimed at identifying when the IMU is stationary, so that the ZUPT can be applied, have been proposed in [40]. These detectors are all generalized likelihood ratio tests; they differ in the assumptions of prior knowledge about signals dynamics, under the hypothesis that the IMU is stationary. As shown in [40], the methodological differences among different ZUPT interval detectors may lead to double the errors in position estimation. The angular rate energy (ARE) detector [40] is computationally one of the lightest and provides the highest position accuracies; and, it is one of the most robust to changes in gait speed.

Based on these characteristics, ARE was selected as the ZUPT interval detector to be used in the system. In addition to the statistical analysis of the angular rate signal, an innovative module exploiting IC and FO time and amplitude knowledge was introduced in order to improve the specificity of the identification of stationary states. In particular, within each interval lasting from an FO to following IC, it was imposed to not have ZUPT occurrence. Furthermore, the standard deviation of gyroscope noise used to control the ARE trust in the gyroscope data, as well as the threshold and the window size used to determine the ZUPT occurrence are automatically tuned by the algorithm on the basis of the IC and FO amplitudes.

As a real-time application, gait events, ZUPT interval identification and signal filtering are executed in a continuous way as soon as new measurements are available, allowing the tracking of movements in real time (see Section 4.4 for the method accuracy).

### 4.3. Trunk Flexion and Gait Asymmetry Estimation

Gait asymmetry was obtained by applying the equation provided in [41]. Trunk flexion was simply obtained as the inverse of the sinus of the anteroposterior component of the acceleration signal, provided from the sensor attached to the trunk. Similarly to other gait parameters, trunk inclination is monitored during the walk, and a vocal request of restoring an upright posture is provided every time the patient bends forward (see Section 4.5).

### 4.4. Step Length Estimation Accuracy in Clinical Settings

**Figure 6.**
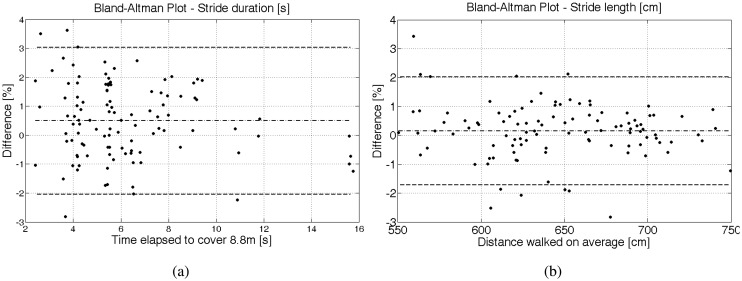
Bland–Altman plots for percentage differences for (**a**) the step duration and (**b**) the step length estimated with inertial measurement units (IMUs) and GAITRite (GR).

In order to test the step length, five male PD patients walked five times at comfortable, twice at increased and twice at decreased gait speed over an 8.80-meter instrumented GAITRite walkway (GR, CIR Systems Inc, PA) set at 100 Hz. The patients were aged 5,087 years with Hoehn and Yahr stages 13. Hoehn and Yahr is a clinical rating scale for disease severity in Parkinson, ranging from zero (no signs of the disease) to five (bedridden). The patients were tested on medication, taken in their usual dosage. The IMUs were set at 200 Hz and fastened on top of the participants' shoes; the same two IMUs with the same Kalman filter settings were used for all patients. IMU data collection was performed via Bluetooth by means of the Android application. For each of the nine trials, the total duration and length of the strides was obtained both from IMUs and GR. Figure 6 shows, in Bland-Altman plots, the differences in the percentage between step duration and length estimated with IMUs and GR.

The results obtained show a good agreement between the INSand GR method, with a bias close to zero and an SD within 3% for stride duration and 2% for stride length. The performance obtained among the five subjects and the two sides (that is, two IMUs) is consistent, revealing a good inter-subject and inter-hardware reliability. A single side of one patient produced accelerations above the full scale of the IMUs (±6 g), revealing the requirement of setting the acceleration full scale higher than 6 g.

In conclusion, ZUPT-aided INS by means of foot-mounted IMUs is robust and accurate enough to estimate step length over intervals of a mid-range distance on patients with PD.

### 4.5. Real-Time Audio Feedback Application

In order to monitor and guide the patient performance during the gait, the system computes in real time the key gait parameters and relates them with respect to a reference, providing a vocal request for changing the gait pattern every time the match is not satisfying or a vocal reinforcement every time it is.

**Figure 7.**
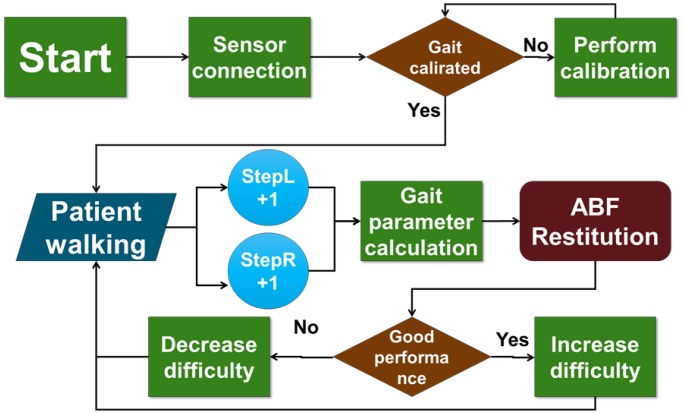
Block diagram of the audio feedback application.

The system architecture is schematized in Figure 7. This architecture has been implemented into an Android standalone application, which processes data streamed from the sensor nodes via Bluetooth. It was tested on different dual core smartphones, always achieving real-time performance and running with no delay. The application was evaluated on an LG Nexus 5 phone, where the time needed to perform the algorithm was measured. The operations executed at each new sample and at each new step detected represent the main computational load of the algorithm, and they are executed in 0.11 *ms* and 0.23 *ms*, respectively. This computational load guarantees the real-time execution of the application with no delays.

Considering the huge variability among the gait patterns of PD patients, the reference values cannot be the same for each patient, but should be tailored to specific subject's needs, taking into account the actual patient's status and clinical expectations. For this reason, the system includes a function able to compute a subject-specific calibration. During this calibration phase, the patient is asked to walk about 20 to 40 m under the supervision of the clinician. In this session, the clinician assesses the patient's gait pattern and gives instructions on how to improve aspects of the gait (e.g., take large steps). When the clinician is satisfied with the improvements made by the patient, the trial data is stored into the system and establishes the target (zone) for following gait trainings. It is the clinician's knowledge and patient's locomotor evaluation that determine what gait pattern and overall performance the patient should provide during this calibration trial. In this way, the system stores a customized target that the patient should commit to maintaining along the following daily practice.

Conceptually, the vocal requests of changing the pattern are fed back to the patient every time the (mean between right and left sides) percentage difference of the current value with respect to the reference is above an upper tolerance or below a lower one. Upper and lower tolerances represent unitless parameters, theoretically variable from 0% up to 100%, that is, respectively, complete or absent adherence to the reference. The resulting confidence interval allows the system customization on the basis of patient motor abilities. Once the patient is able to perform within this interval, a vocal reinforcement is played.

Noteworthy is the system being able to automatically increase the difficulty of the task to follow a patient's performance. In particular, every time the patient is able to remain within the confidence interval for a certain amount of steps (set by the clinician), the upper and lower tolerances are progressively decreased to a certain extent (set by the clinician), providing a more challenging exercise. Moreover, the system is able to adjust the feedback messages in order to be compliant with patient preferences. In particular, in order to avoid an annoying and/or distracting effect, two solutions were adopted. First, each vocal message provided to patients is randomly selected from three different mp3 files, each containing a different expression of the same content. Second, as long as the patient is able to provide a constant performance, either remaining within the confidence interval or out of it, such that the content of the message is the same, the amount of vocal feedbacks returned to the patient is progressively reduced.

### 4.6. Proposed Usage Scenario

The typical scenario of the use of the system entails the patient walking freely outdoors, in a park, for example, over single or multiple periods of 30 min each. During a training session, the system measures the selected gait parameters and compares them with respect to reference values over windows of some steps (e.g., every five steps).

## 5. Discussion and Outlook

In this work, we presented a system for gait training to be used in a daily life setting, based on a fully portable and wearable solution and able to provide real-time audio feedback by means of vocal messages. The system is now being used for pilot trials with PD patients, and results will be employed to adjust the application for a more extensive clinical usage. A potential advantage of the system is the possible continuous training for a long period of time, a key element in the rehabilitation of a chronic disease, such as PD. In fact, long-term treatment in a clinical setting is hardly sustainable and is cost ineffective.

In addition, the gait features computed by the system may also be exploited in the future as methods to assess gait, both in a laboratory setting (as in the traditional context of gait analysis) and in an out-of-the lab environment (e.g., in the home, but also during clinical routines). In this last case, in particular, the present approach based on a few portable sensors delivers its major potential. To this aim, further analysis is desirable, to validate the spatio-temporal feature estimation.

To fully exploit the leverage of the application for gait training, some further steps are necessary to optimize the context awareness of the system and disambiguate, for example, if the user's gait presents bradykinesia or even freezing of the gait, or if she/he is simply distracted or stopped by events happening during the exercise.

Thanks to the logging capability of the system, and the possibility of evaluating the overall performance immediately after the training, it is possible to monitor the state of the user over a number of days, in an automatic manner. In fact, all the gait features, the overall performance, the ability to follow the feedback and to correct the gait, are stored in the smartphone and can be evaluated after the treatment or even after sessions of treatment to study the response of the patients. Furthermore, adherence to therapy may be automatically evaluated (it is possible to understand if a user performed the training or not), and this, indeed, presents a significant benefit for the clinician following the patient.

The system is suitable to be used on different populations other than PD, where gait may be trained according to the specific features considered in the present study. In particular, in cases of stroke rehabilitation or in the case of orthopedic impairments, the system may help in restoring a physiological gait pattern. In addition, with further developments, such an approach may be a tool for sports applications. Thanks to the ability to track the user's path, it can complement the efficacy of a localization system.

Further studies are foreseen to deeply evaluate the kind of feedback provided to the user. In this case, it was decided to provide therapist-like messages. It is also possible to provide RAS, an alarming function, music or other sound modalities to the user, depending on the requirements of the actual gait pattern.

From a technology point of view, the discussion of using the smartphone is still open. From one side, the user wants the number of sensors and devices to be minimized. From another point of view, the smartphone is a powerful platform, with various interfaces and sensors, enabling geo-localization and allowing the development of simplified interfaces for non-expert users. However, it is still another device to be recharged and maintained. That is why we are evaluating embedding and optimizing the algorithms in the nodes themselves, eventually distributing the processing and specializing the nodes. This process may, on the other side, limit the level of generalization and the adaptability of the system, which is now possible using smartphones. A trade-off between simplification and reconfigurability remains, in fact, one of the main challenges for the acceptance of technology in non-specific fields.

## Figures and Tables

**Table 1.** Gait features and the corresponding instruction to be fed back to the user in real time and in a closed loop by the wearable sensors and system.

**Gait Features**	**Content of the Instruction to the User**
Cadence (number of steps per minute)	increase/decrease gait speed
Step length	keep steps longer/shorter
Gait speed	increase/decrease gait speed
Gait asymmetry	increase right/left step length
Trunk flexion	keep upright posture
Clearance	rise right/left leg

**Table 2.** Static noise estimation for the different sensors of the EXEL and Xsens sensing nodes.

	**EXLs1**			**MTw**		

	**X**	**Y**	**Z**	**X**	**Y**	**Z**
Accelerometer	0.0034	0.0019	0.0204	0.0156	0.0126	0.0325
Gyroscope	0.0103	0.0034	0.0047	0.0049	0.0055	0.0054
Magnetometer	0.0079	0.0077	0.0220	0.0014	0.0013	0.0023
